# Effects of Solute-Solute Interactions on Protein Stability Studied Using Various Counterions and Dendrimers

**DOI:** 10.1371/journal.pone.0027665

**Published:** 2011-11-18

**Authors:** Curtiss P. Schneider, Diwakar Shukla, Bernhardt L. Trout

**Affiliations:** Department of Chemical Engineering, Massachusetts Institute of Technology, Cambridge, Massachusetts, United States of America; Consejo Superior de Investigaciones Cientificas, Spain

## Abstract

Much work has been performed on understanding the effects of additives on protein thermodynamics and degradation kinetics, in particular addressing the Hofmeister series and other broad empirical phenomena. Little attention, however, has been paid to the effect of additive-additive interactions on proteins. Our group and others have recently shown that such interactions can actually govern protein events, such as aggregation. Here we use dendrimers, which have the advantage that both size and surface chemical groups can be changed and therein studied independently. Dendrimers are a relatively new and broad class of materials which have been demonstrated useful in biological and therapeutic applications, such as drug delivery, perturbing amyloid formation, etc. Guanidinium modified dendrimers pose an interesting case given that guanidinium can form multiple attractive hydrogen bonds with either a protein surface or other components in solution, such as hydrogen bond accepting counterions. Here we present a study which shows that the behavior of such macromolecule species (modified PAMAM dendrimers) is governed by intra-solvent interactions. Attractive guanidinium-anion interactions seem to cause clustering in solution, which inhibits cooperative binding to the protein surface but at the same time, significantly suppresses nonnative aggregation.

## Introduction

Understanding how solution components interact with proteins and modulate biological processes is essential in applications such as researching methods for stabilizing protein based therapeutics and treating neurodegenerative diseases resulting from amyloid formation [1]. Dendrimers are a relatively new class of materials with much research already devoted toward possible biomedical applications, which is quite promising [2]–[5]. The interaction of dendrimers with proteins is a classic example of polyvalent interactions, which is a common form of interaction between biological entities, such as the interaction between receptors and ligands, the interaction between a virus and a cell surface, etc. [6]. Such interactions lead to a varying array of resulting effects on protein behavior. In certain cases, polycationic dendrimer molecules destabilize protein conformations [7], [8] and enhance the formation of amorphous aggregates [9]. However, the same dendrimer species have also been shown to dissolve amyloid fibrils and inhibit their formation [9]. While in other cases, strong adhesive interactions with oxyanionic groups in proteins stabilize protein assemblies, such as microtubules [10]. In several of these cases, derivative dendrimer species were produced by modifying the surface to guanidinium [8], [11], a functional group known to preferentially bind to protein surfaces [12].

When considering the behavior of solution additives or macromolecular species with charged surfaces, the influence of the counterion is often overlooked. The influence individual ions have on protein stability has been empirically ranked for more than a century in the well-known Hofmeister Series [13] and correlated with respect to preferential interactions [14]. Furthermore, it has long been believed that the net effect of a salt is the additive effect of each ion [15]. However, recent investigations suggest that for the particular case of guanidinium bearing compounds, the strength of attractive ion-ion interactions are the cause of varying behavior among different guanidinium salt forms [15]–[20]. This model explains the neutral behavior of guanidinium sulfate without the need for an unfavorable separation of charge [15]. That is, rather than a bound guanidinium cation and an excluded sulfate anion, the attractive interaction between guanidinium and sulfate causes clustering, which interferes with the binding of guanidinium to the protein surface. More importantly, such interactions will be of particular importance for compounds containing multiple guanidinium groups, such as the previously mentioned dendrimers. If the net effect of combining ions is purely an additive effect, then exchanging the counterion should not change direct protein-dendrimer interactions or the behavior of dendrimer molecules in solution. However, if the recently revealed interactions are correct, exchanging the counterion will significantly alter the behavior of the guanidinium compounds as the effects of ion-ion interactions will be amplified.

Here, we present a study which shows that attractive guanidinium-anion interactions strongly influence the solution behavior of guanidinium modified PAMAM dendrimers. As with other similar compounds, the guanidinium chloride (GdmCl) form disrupted attractive protein-protein interactions at low concentrations but reduced thermostability, which led to enhanced aggregation. However, the aggregation suppression by the sulfate and dihydrogen phosphate salt forms was more significant and observed at all concentrations. They slowed the rate of aggregation of model proteins (α-Chymotrypsinogen A and Concanavalin A) to about 2% of the original aggregation rate at concentrations as low as 0.2 mol/L, which is around 10 times slower than when in the presence of arginine HCl or other aggregation suppressing excipients (e.g. sucrose, glycerol, etc.). Preferential interaction and computational studies of the modified generation 0 dendrimer salts in α-Chymotrypsinogen A aqueous solutions establish that attractive ion-ion interactions alter how the dendrimers interact with each other and with proteins through the formation of clusters. Such behavior is also observed for the unmodified ammonium surface but to a much lesser degree [21], demonstrating the specific nature of the attractive ion-ion interactions. Understanding the solute-solute interactions presented in this study gives valuable insight into the overall understanding of how ion interactions influence the behavior of macromolecular compounds with polycationic surfaces.

## Results

### Aggregation Suppression

**Figure 1:**
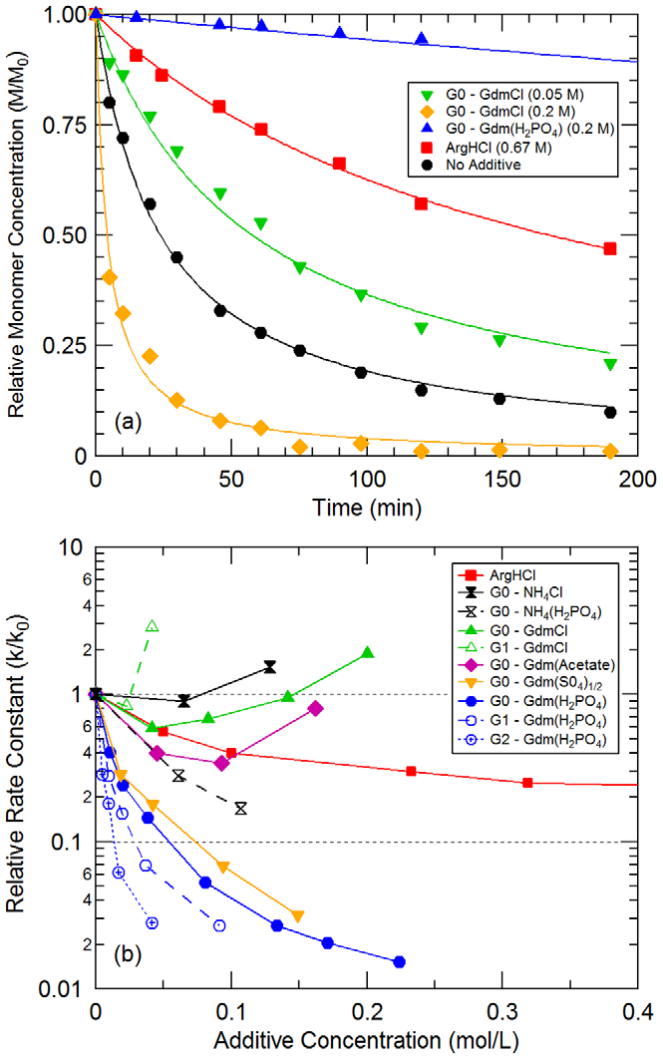
The influence of guanidinium modified PAMAM dendrimers on aCgn monomer loss due to aggregation. For all experiments, samples were incubated at 52.5°C, initial monomer concentration, M_0_, was 10 mg/mL, all solutions were prepared in a 20 mM sodium citrate pH 5 buffer, and all rate loss profiles fitted to a 2^nd^ order rate law. (a) Monomer loss profiles for solutions containing Generation 0 PAMAM dendrimers with guanidinium chloride or H_2_PO_4_ surfaces at varying concentrations. (b) Rate constant, k, for aCgn monomer loss relative to the rate constant for no additive, k_0_, versus additive concentration.

The most notable consequence of perturbing a protein's environment is the enhancement or inhibition of protein aggregation [22], [23]. The guanidinium modified dendrimers were added to model protein solutions and incubated at an elevated temperature to determine how they influence the rate of this degradation pathway. Figure 1a shows α-chymotrypsinogen A (aCgn) monomer loss profiles, as determined by size exclusion HPLC, for solutions containing a generation 0 PAMAM dendrimer with the surface modified to GdmCl. At low dendrimer concentrations (0.05 M), the rate of monomer loss in the presence of the surface modified dendrimer is slower than when compared to the reference solution. However, this aggregation rate reduction is insignificant when compared to the rate reduction induced by a high concentration of other commonly used additives such as arginine hydrochloride (ArgHCl), which is also depicted in the figure. Furthermore, as the concentration of the surface modified dendrimer is increased, the aggregation rate reduction decreases until ultimately, the rate of aggregation is increased. At a concentration of 0.2 M, the surface modified dendrimer induces rapid aggregation, causing a 50% loss in about 10 minutes as opposed to 30 minutes for the solution containing no cosolute. These results only become worse for higher generations. As shown in Figure 1b, the rate of monomer loss for the generation 1 dendrimer with a surface modified to GdmCl indicates a large increase in the rate of aggregation at concentrations as low as 0.05 M, even though at lower concentrations the compound inhibits aggregation by a moderate amount.

These results are comparable to other large compounds with surfaces modified to Gdm, which exhibit a strong interaction with proteins that results in destabilization at moderate to high concentrations [8], [10]. However, a previous inquiry into various arginine salts [20] showed that the interaction between a Gdm functional group and a protein is strongly influenced by the counterion to the Gdm moiety. The reason for this is that hydrogen bond accepting anions will tend to form strong hydrogen bonds with the hydrogen bond donating Gdm group. Thus the interaction between the modified dendrimers and the protein can be altered by exchanging chloride with counterions such as sulfate, phosphate, citrate, acetate, etc., which are more capable of accepting hydrogen bonds [16].

The results shown in Figure 1a reveal that a generation 0 PAMAM dendrimer with a surface modified to guanidinium dihydrogen phosphate (H_2_PO_4_) has an ability to slow the rate of aCgn aggregation far beyond that of ArgHCl. To elaborate, with no cosolute present, half of the original amount of protein is lost within only 30 minutes. When in the presence of a high concentration of ArgHCl (0.67 M), the half-life is extended to about 2.5 hours, which is similar to other commonly used excipients. However, when in the presence of the H_2_PO_4_ form of the surface modified dendrimer at a concentration of 0.2 M, the half-life is extended to about 25 hours (see Figure S1 which shows extended data), which is a full order of magnitude longer than the solution containing ArgHCl. This superior aggregation suppression is observed at all concentrations for that salt form, as shown in Figure 1b. In that figure, the relative rate constant for aCgn monomer loss is depicted, which is the observed rate constant when a cosolute is added to the solution relative to the rate of monomer loss in a buffer only solution. The figure also reveals that aggregation suppression improves with increasing size of the dendrimer, as indicated by the monotonic improvement in aggregation suppression with each dendrimer generation.

Improved aggregation suppression is also observed when the counterion is exchanged to other hydrogen bond accepting anions. When exchanged to acetate, aggregation suppression is only improved slightly, which was anticipated given that acetate cannot form as many hydrogen bonds with Gdm as compared to other ions [20]. When chloride is exchanged with sulfate though, the resulting compound shows nearly identical ability for inhibiting aggregation as the H_2_PO_4_ salt form. The sharp decline in the rate of aCgn aggregation prompted us to study dendrimer-aCgn interactions, both experimentally and computationally, to give greater mechanistic insight into the observed behavior. However, we first expanded the aggregation study to determine if the observations are observed for other proteins. Concanavalin A (Con A) was used as another model protein for the aggregation study, which demonstrated that the shelf-life of this protein at pH 6.5 and 37°C is extended by a factor greater than 15 when in the presence of the generation 1 dendrimer with a guanidinium sulfate surface (see Table 1), which is over 10 times longer than when in the presence of commonly used additives such as glycerol or sucrose. Table 1 depicts the factor by which the shelf-life of aCgn and Con A is extended when they are formulated with the modified dendrimers. This Shelf-Life Extension Factor was determined by comparing the length of time, t_95_, for a 5% loss of protein when in the presence of the compounds to the original length of time, t_95,0_ for a 5% loss. For comparison purposes, isotonic concentrations (as determined by VPO) of the compounds were used and Shelf-Life Extension Factor values for commonly used excipients at isotonic concentrations are shown as well. Shelf-life values of aCgn were determined at 52.5°C and at 37°C for Con A. It is clear from these results, that when utilized at a practical maximum concentration, the surface modified dendrimers, in the form of either a dihydrogen phosphate or sulfate salt, significantly improve the shelf life of these two proteins, either at high or moderate temperatures. For aCgn, the shelf life is extended by a factor between 16 and 27 when formulated with these dendrimers, which is 5 to 8 times longer than when in the presence of other aggregation suppressing additives, such as arginine HCl, sucrose, or sodium sulfate.

**Table 1:** Protein solution shelf-life extension at accelerated conditions resulting from aggregation suppression induced by surface modified PAMAM dendrimers and other commonly used additives formulated at isotonic concentrations.

Additive	Gen.	Surface	Conc.	aCgn	Con A
			mM	t_95_/t_95,0_	t_95_/t_95,0_
Sucrose	-	-	280	1.9	1.5
Glycerol	-	-	280	-	1.3
Na_2_SO_4_	-	-	140	3.1	1.1
ArgHCl	-	-	170	3.3	0.4
Dend.	0	Gdm(SO_4_)_1/2_	140	26.9	5.9
Dend.	0	Gdm(H_2_PO_4_)	80	18.9	1.6
Dend.	1	Gdm(SO_4_)_1/2_	70	-	16.7
Dend.	1	Gdm(H_2_PO_4_)	42	16.3	-

The aCgn solution was formulated in a 20 mM sodium citrate pH 5 buffer and was incubated at 52.5°C. The Con A solution was formulated in a 40 mM sodium phosphate pH 6.5 buffer and was incubated at 37°C.

The results for Con A are more significant and show a much clearer relationship with the size of the dendrimers. At 37°C and pH 6.5, Con A aggregates quite rapidly. Commonly used additives can extend the shelf life, at most, by factor of only 1.5. The sulfate form of the generation 0 modified dendrimer can quadruple this to a factor of 5.9 and the sulfate form of the generation 1 modified dendrimer extends the shelf life even further, by a factor of 16.7. The phosphate form only shows a minimal improvement in the shelf life, likely due to Con A being very sensitive to ionic strength at pH 6.5 because arginine HCl, sodium chloride, and sodium phosphate all speed up Con A aggregation. The sulfate form of the dendrimers has fewer ions per mole than the phosphate form and therefore, the detrimental effect imparted on proteins that are sensitive to ionic strength is lessened when this form of the dendrimer is used.

It should be noted that these results do not extend completely to the original, unmodified dendrimer structure, which has an ammonium surface. The chloride form of the unmodified dendrimer is more destabilizing and the phosphate form is less effective at suppressing aggregation (see Figure 1b). This indicates that a Gdm surface is a necessity to produce the potent aggregation suppressing results through both protein-additive and ion-ion interactions.

### Conformational Stability

The thermostability of aCgn at 1 mg/mL in the presence of the modified dendrimers was assessed by determining the denaturation midpoint temperature (T_m_) from DSC scans, which is a qualitative indicator of how the conformational stability of the protein is perturbed [24]. As shown in Table 2, the sulfate and H_2_PO_4_ salt forms of the surfaced modified generation 0 dendrimers increase T_m_ at a rate of 15.2 and 37.4°C*M^−1^, respectively, for concentrations less than 0.2 mol/L, while the chloride salt form decreases T_m_ at a rate of 13.9°C*M^−1^. One can speculate that this indicates that the sulfate and H_2_PO_4_ salt forms shift the protein folding equilibrium toward the native structure while the chloride salt form promotes unfolding. However, given that the unfolding of aCgn is irreversible, it could also indicate that the sulfate and H_2_PO_4_ salt forms reduce the rate at which aCgn aggregates during the DSC scan. The apparent thermodynamic stabilization by these forms is quite significant when compared to other conformational stabilizers (e.g. sucrose) [20] given that the results likely represent a combination of conformational stabilization and association suppression. The rate at which the chloride salt form lowers the melting temperature of aCgn is double that for ordinary GdmCl [20] and given that this dendrimer salt form inhibits aggregation at low concentrations, this shows that this surface modified dendrimer is a powerful denaturant.

**Table 2:** Summary of key data for each guanidinium modified PAMAM dendrimer salt, demonstrating their physical properties and their interaction with aCgn.

Surface	MW	V_o_	Γ_23_/ [3]	dT_m_/d [3]	Number of Hydrogen Bonds					
	g/mol	L/mol	(mol/mol)	K*L/mol	D-D	D-A	D-A-D	P-D	P-A	P-A-D
GdmCl	903.6	0.5217	(−8.1±3.6)	−13.9	1	21	1	22	5	0
Gdm(SO_4_)_1/2_	979.1	0.5665	(−17.0±4.4)	15.2	1	146	124	13	13	35
Gdm(H_2_PO_4_)	1272.8	0.7254	(−15.8±5.0)	37.4	5	150	73	14	18	32

MW-molecular weight, V_o_-partial molar volume at infinite dilution, [3]-molar concentration of the additive, D-Dendrimer, A-Anion, and P-Protein. Partial molar volume was determined from density measurements of gravimetrically prepared dendrimer only solutions. Preferential interactions (Γ_23_) with aCgn were determined by VPO, aCgn denaturation midpoint temperature (T_m_) increments were determined by DSC, and the number of hydrogen bonds between different species were determined from MD simulations. aCgn solutions for the Γ_23_ (50 mg/mL) and T_m_ (1 mg/mL) data contained 20 mM sodium citrate pH 5 buffer and a maximum dendrimer concentration of 0.2 mol/L.

### Ion-Ion Interactions

**Figure 2:**
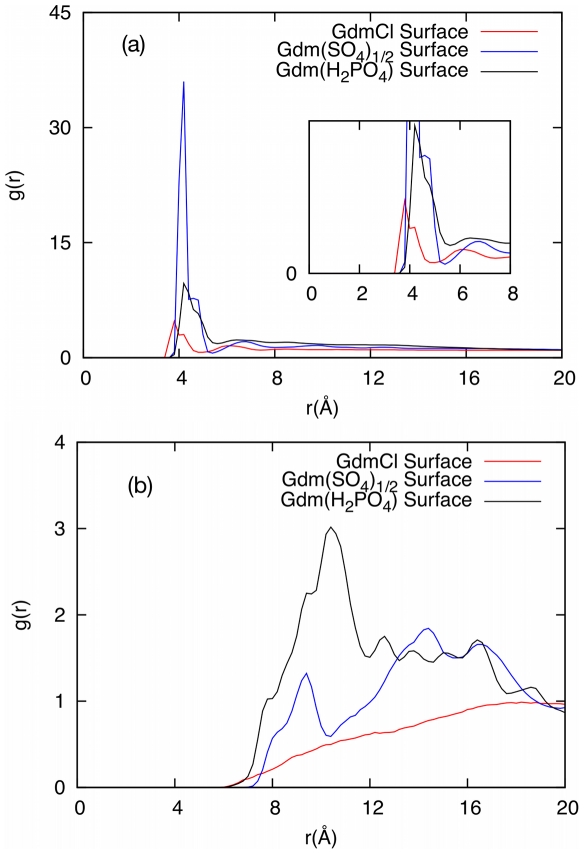
Denderimer Radial Distribution Functions (RDF's). (a) RDF's between dendrimer and counterions and (b) between dendrimer molecules in different dendrimer salt solutions. The distance between the centers of mass of the dendrimers is used for calculation of the RDF's. For the counterions, the sulfur atom in sulfate, phosphorus atom in H_2_PO_4_ are utilized.

**Figure 3:**
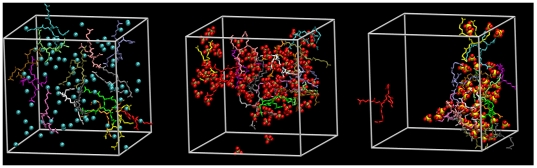
Snapshots of aqueous generation 0 PAMAM dendrimer salt solutions obtained from MD simulations. The counterion is either chloride (left), H_2_PO_4_ (middle) or sulfate (right). The dendrimer molecules are shown in Licorice style and counterions are shown as VdW spheres. The hydrogen atoms are not shown to improve the clarity.

MD simulations were conducted on aqueous solutions of the modified generation 0 dendrimers to quantify how ion-ion interactions may be influencing the behavior of the additives (see Table S1 for a description of the setup of each simulation). In Figure 2a, the Radial Distribution Functions (RDF) between the dendrimer and the counterions show that the sulfate and H_2_PO_4_ ions interact strongly with the dendrimer molecules, as shown by the height of the peaks relative to chloride. In Figure 2b, the RDF's between dendrimer molecules indicate that in the presence of chloride ions, dendrimer molecules do not interact with each other, however, the presence of sulfate and H_2_PO_4_ ions tends to bring dendrimer molecules together. This is further supported by MD snapshots of the simulation box (Figure 3), which show significant ion pairing in the sulfate and H_2_PO_4_ salt solutions while solutes in the chloride solution are randomly distributed. These results indicate that the Gdm group on the dendrimers can form charge-assisted hydrogen bonds with the sulfate and H_2_PO_4_ ions. The sulfate ion, which has a −2 charge on four oxygens, forms a much stronger hydrogen bond as compared to the H_2_PO_4_, which has −1 charge.

These results also show that counterions can act as a bridge between dendrimer molecules due to attractive guanidinium-anion interactions, leading to the formation of large clusters in solution (see Figure 3). To verify and quantify this observation, the number of hydrogen bonds formed between different ion-pairs in aqueous modified dendrimer salt solutions was calculated from the simulation results (see Table 2). Sulfate and H_2_PO_4_ ions, due to the presence of multiple hydrogen bond donors and acceptors, indeed act as a bridge joining dendrimer molecules together. The number of hydrogen bonds for both salt types (∼150) is nearly an order of magnitude more than that for the chloride form (∼20), leading to numerous bridged interactions (73 to 124), which is almost nonexistent for the chloride form. These guanidinium-anion and bridged interactions have a direct impact on the number of dendrimer-protein interactions, reducing the number by nearly half when compared to the chloride form. The extent of clustering in these solutions can also be quantified in terms of the loss of the solvent-accessible area (SAA) of dendrimer molecules, as shown in Table 3. The loss of SAA due to clustering is greatest for H_2_PO_4_ (∼60%), followed by sulfate (∼40%) and chloride (20%). In the case of chloride, the loss of SAA is mainly due to the presence of counterions near the dendrimer. For sulfate and H_2_PO_4_, the dominant component to the loss of SAA is due to the overlap of dendrimer molecules. The number of H_2_PO_4_ ions is twice the number of sulfate ions per dendrimer molecule, which contributes to the higher loss of SAA as compared to sulfate.

**Table 3:** Loss of solvent-accessible surface area (SAA) of modified generation 0 PAMAM dendrimers due to clustering in aqueous solutions.

Surface	SAAÅ^2^	ΔSAAÅ^2^	ΔSAA (Å^2^)dendrimeroverlap	ΔSAA(Å^2^)counter-ionoverlap
GdmCl	993	267	107	160
Gdm(SO_4_)_1/2_	760	500	342	158
Gdm(H_2_PO_4_)	533	727	435	292

The SAA of a dendrimer molecule in water is 1260 Å^2^.

### Preferential Interactions

To gain insight into how the modified dendrimer salts inhibit protein-protein interactions, preferential interaction coefficient, Γ_23_, values at various concentrations were determined, both experimentally via vapor pressure osmometry (VPO) measurements, and computationally via MD simulations. The experimental results for the interaction between modified generation 0 PAMAM dendrimers and aCgn are expressed in Table 2, which summarizes the polynomial fit and uncertainty of the experimental data. Theoretical preferential interaction coefficient values were computed from the MD simulation (see Figure S2, which depicts the convergence of simulated values) using the procedure outlined in our previous work [25], [26] and the results are presented in Table 4.

**Table 4:** Preferential interaction coefficient values of α-Chymotrypsinogen A in aqueous modified generation 0 PAMAM dendrimer solutions.

Surface	Conc.(mol/L)	Γ_exp_	Γ_MD_	Γ_MD_Dend.	Γ_MD_Anion
GdmCl	0.18	−1.5	−0.2	1	−7
Gdm(SO_4_)_1/2_	0.18	−3.1	−2.7	−3	−7
Gdm(H_2_PO_4_)	0.17	−2.9	−2.3	−3	−10

Standard deviations on the preferential interaction coefficient values are ∼1.

**Figure 4:**
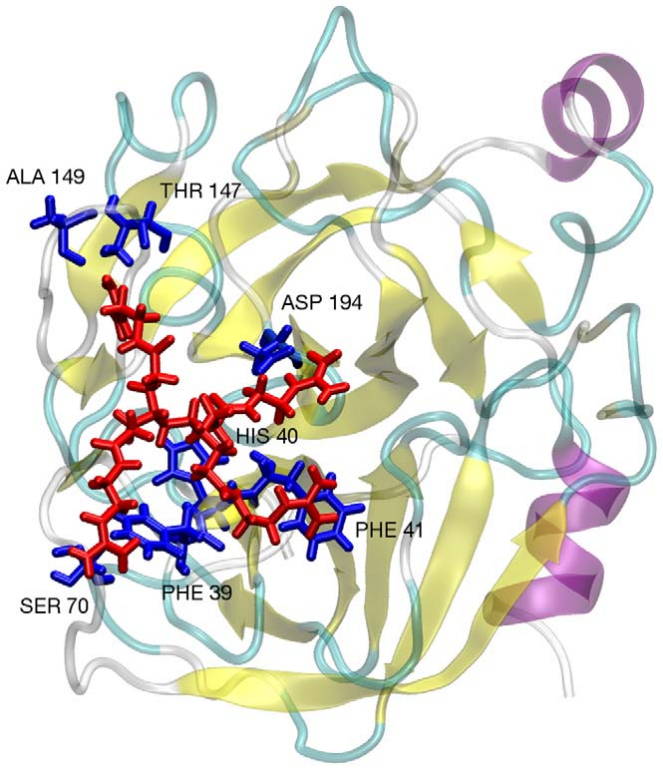
PAMAM dendrimer with guanidinium chloride surface interacting with multiple groups on the surface of aCgn. The guanidinium groups can hydrogen bond with negatively charged amino acids and the peptide backbone. They can also form cation-π interaction with aromatic amino acids.

At a concentration of 0.18 mol/L, the theoretical preferential interaction coefficient for the chloride salt is found to be −0.2±1, which matches well with the experimental value of −1.5±0.7. The Γ_23_ values for salts are a weighted average of the Γ_23_ values for individual ions. Γ_23_ for the dendrimer cation was found to have a positive value of 1, which shows that the local concentration of dendrimer molecules around the protein is higher than the bulk concentration. However, due to the negative preferential interaction value for the chloride ion (−7), the overall preferential interaction coefficient was found to be negative. The observed preferential binding of the dendrimer cation stems from the fact that the modified dendrimers can interact favorably with a variety of amino acids on the protein surface due to the presence of the Gdm group, which can form hydrogen bonds with negatively charged amino acids and the protein backbone and can also interact with aromatic amino acids via cation-π interactions. Furthermore, the dendrimer molecule can bind cooperatively with the protein surface due to multiple Gdm surface groups simultaneously interacting with the protein surface (see Figure 4, which shows a snapshot of multiple, simultaneous interactions). However, switching the counterion to either sulfate or H_2_PO_4_ inhibits the occurrence of such multiple interactions.

Γ_23_ values for the sulfate (−2.7) and H_2_PO_4_ (−2.3) salt forms match well with their corresponding experimental values. The values of Γ_23_ for the counterions are −10 for the H_2_PO_4_ ion and −7 for the sulfate ion, which is present in half the quantity as the phosphate and chloride ions. On the basis of the observed attractive interaction between the dendrimer and these counterions, it can be argued that sulfate and H_2_PO_4_ inhibit the dendrimer molecule from binding to the protein surface. In essence, the dendrimer molecules are pulled away from the surface to interact with bulk solution components. This is verified by the individual Γ_23_ values for the dendrimer molecule (−3 for both salt types). These results are similar to the results of our recent work on the interaction of arginine with proteins, where the carboxylate group and various counterions limited the interaction between a protein and the Gdm group in arginine [18]–[20]. As mentioned before, the reduced number of hydrogen bonds between the protein and the dendrimer (see Table 2) further supports this behavior. The loss in the number of direct hydrogen bonds is compensated by the increase in the number of indirect hydrogen bonds formed between the protein and the dendrimer in which the counterion acts as a bridge.

**Figure 5:**
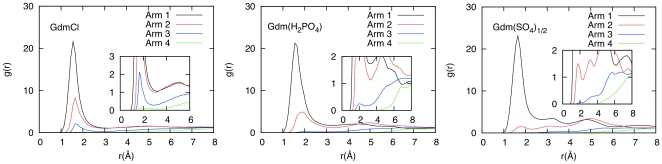
RDF's between α-Chymotrypsinogen A and the surface guanidinium groups on the PAMAM dendrimer. The counterion is either chloride (left), H_2_PO_4_ (middle) or sulfate (right). The arms of the dendrimer are labeled 1–4 depending on their distance from the protein surface, with 1 denoting the closest arm. The distance of the central carbon atom in the guanidinium group from the protein surface is used for the calculations.

RDF's between the four dendrimer arms and the protein surface (see Figure 5) highlight the implications of the counterions interacting with the Gdm groups. The RDF for the closest arm remains almost the same for all dendrimer salts but the RDF's for the remaining arms show a sharp decrease in peak height and increased distance from the surface of the protein for the sulfate and H_2_PO_4_ salt forms. This result further supports that for the dendrimer with a GdmCl surface, multiple arms simultaneously interact with the protein surface but for the sulfate and H_2_PO_4_ salts, only one arm can interact with the protein while the other arms face away from the surface and interact with the bulk solution. Furthermore, for the sulfate and H_2_PO_4_ salts, there are additional peaks further away from the surface for the closest dendrimer arm, which is the result of the anions acting as a bridge between the protein and the dendrimer. This interaction with the Gdm group is clearly impeding direct binding of the dendrimer to the protein surface.

## Discussion

The original intent of this work was to determine if attractive protein-protein interactions could be inhibited by large additives which tend to crowd the local domain around proteins rather than being excluded to the bulk solution [27]. It was theorized that the crowding could be promoted by counteracting repulsive steric exclusion interactions (which are significant for large additives) with the attractive interactions that occur between a protein and a denaturant. That is, creating a balance of attractive and repulsive interactions that lead to a net-neutral interaction. If such a balance occurs, the resulting compound would interfere with protein-protein interactions with little influence on conformational stability [27], [28]. Given the suppression of protein aggregation at low concentrations, the results presented here show that large compounds (i.e. dendrimers) with protein-binding functional groups (i.e. GdmCl) on their surfaces disrupt protein-protein interactions due to an attractive interaction with the protein. However, for the particular case of guanidinium chloride modified PAMAM dendrimers, the net attractive interaction seems to be too strong given the conformational destabilization and enhanced aggregation at higher concentrations. The compounds can inhibit aggregation at a level comparable to other commonly used excipients but only at low concentrations.

From these results, it is obvious that volume exclusion effects (see Table 2 and Table S2, which give values for the molecular weight and partial molar volume of the modified dendrimers, which are larger by several factors than most small molecule additives) are not counteracting the preferential binding of the surface groups to the extent anticipated. Preferential binding is predicted to scale with the number of binding groups per area in accordance with the frequency of single binding interactions with the protein surface, while exclusion is known to scale with the volume of the additive [27]. Thus, if the size of an additive increases while the density of surface groups remains constant, it was predicted that steric exclusion would dominate. However, this does not take into account the total energy of binding nor structural flexibility, which can enhance the density of surface groups. As demonstrated by “molecular glue” compounds [10], which also have multiple Gdm surface groups, the larger and the more flexible the compound, the stronger it binds to proteins. This indicates that the large and flexible nature of the surface modified dendrimers allows for a cooperative interaction of the multiple Gdm groups with the surface of the model protein, which we verified through MD simulations. This attractive interaction is likely stronger for the unfolded state, when more binding sites are exposed and the positive electrostatic charge on the protein surface is distributed over a larger area, enhancing the amount of preferential binding and thus denaturing the protein.

**Figure 6:**
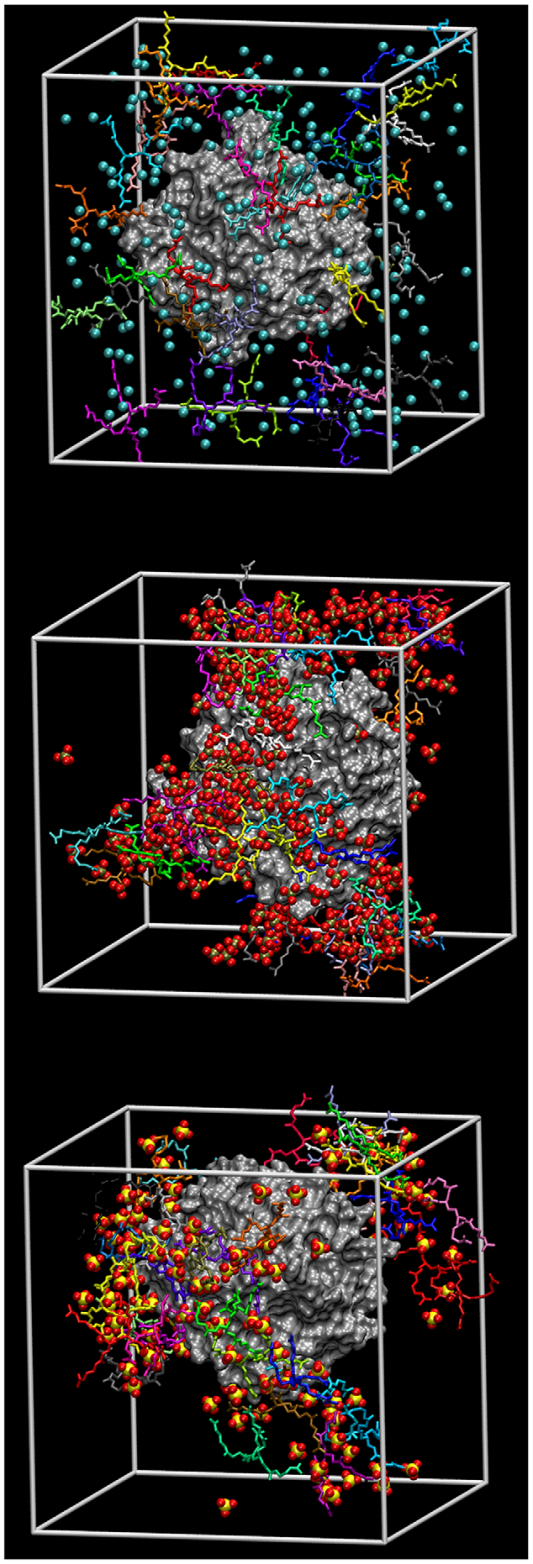
Snapshots of the simulation box from MD simulations of α-Chymotrypsinogen A in the presence of aqueous dendrimer salt solutions. Water molecules and hydrogen atoms are not shown to improve clarity. Dendrimer molecules are shown in licorice representation and counterions are shown in van der Waals representation.

However, as demonstrated by the stabilizing effect and hydrogen bond interactions of the H_2_PO_4_ and sulfate salt forms, ion-ion interactions between the Gdm functional groups and the counterions influence how surface modified dendrimers interact with proteins, in addition to how dendrimer molecules interact with each other in solution. The RDF results show that for these alternate salt forms, the interaction between the protein and the dendrimer is determined by the frequency of single guanidinium-protein interactions (i.e. a single strong guanidinium-protein peak), rather than multiple simultaneous interactions (i.e. multiple strong guanidinium-protein peaks), a behavior more consistent with the predicted outcome of making large compounds with protein-binding groups. The ion-ion interactions described here have only recently been taken into consideration in describing the solution behavior of different guanidinium salt forms. The MD simulations performed verify that attractive ion-ion interactions bridge together multiple dendrimer molecules into clusters. This clustering leads to three important impications: (i) these clusters enhance the effective size of the additives in solution, with the size of the additive determining its ability to crowd out protein-protein interactions, (ii) these clusters are expected to reduce the mobility of the proteins in solutions due to a network of large hydrogen-bonded clusters around them (see Figure 6, which shows snapshots of aCgn in the different dendrimer solutions) which should reduce the rate of protein-protein encounters [29], and (iii) the formation of these clusters influences the interaction between protein and dendrimer molecules. It can be observed directly from the simulations that the cooperative binding of multiple dendrimer arms to the protein surface is inhibited by the cluster formation. This has a direct result in reducing the preferential interaction of the dendrimer molecule and eliminating the denaturing effect.

The trends discussed here are related to the impact these salts have on protein aggregation. Theoretical preferential interaction coefficient values for an inert compound the same size as the modified generation 0 dendrimer but lacking any ability to form attractive interactions shows a preferential exclusion over four times greater than the modified dendrimers (see Figure S3). It is clear from these results that even for the sulfate and H_2_PO_4_ salt forms, the surface modified dendrimers can be considered to be only slightly excluded when compared to how excluded they would be without any protein-binding surface groups. From this perspective, the preferential interaction of the surface modified dendrimers can be considered approximately net-neutral. Also considering that all of the salt forms inhibit protein aggregation at low additive concentrations, such results support the hypothesis that surface modified dendrimers are able to inhibit aggregation, in part, by slowing protein association through a disruption of protein-protein interactions. A highly excluded compound would not exhibit much of an effect on association due to a depletion of cosolute molecules in the local domain of the protein. In fact, large and highly excluded compounds often induce association due to a colloidal depletion force [30], [31]. These results indicate that the counterion plays a critical role in fine tuning the attraction between protein and additive molecules, such that the extent of binding of the modified dendrimer molecule is different among the different salt forms and in certain cases, the attractive interaction between the additives and a protein is strong enough to inhibit protein-protein interactions but not strong enough to denature the protein. This is supported by Figure S4, which depicts a close-up view (within 0.6 nm) of the protein surface showing that the guanidinium chloride modified dendrimers bind to the surface of the protein with no guanidinium-chloride interaction while the guanidinium sulfate modified dendrimers crowd around the protein surface with little binding due to an interaction with sulfate. The exact contribution of inhibiting protein-protein interactions cannot be determined given that the sulfate and H_2_PO_4_ salt forms may also provide conformational stability. However, the sharp decline in the rate of aggregation at low dendrimer concentrations suggests it is a significant contribution, which is further supported by the order of magnitude improvement in the reduction of aggregation when compared to other conformational stabilizers, such as sucrose.

In conclusion, using α-Chymotrypsinogen and Concanavalin A as a model proteins, we investigated the aggregation suppressing performance of PAMAM dendrimers with surfaces modified to a variety of guanidinium salts and give a molecular level mechanistic insight into the behavior of this new class of additive. The most significant observation was that attractive additive-additive interactions dominated the behavior of the dendrimer molecules. The results presented indicate that all of the dendrimers form an attractive interaction with aCgn, leading to suppressed protein-protein interactions, which is more significant than other additives due to the size of the dendrimer molecules. The dendrimers with guanidinium chloride surfaces suppressed aggregation at low concentrations but DSC scans indicate that the additive promotes aCgn unfolding, leading to enhanced aggregation at high concentrations. Under conditions when the conformation of aCgn is not destabilized (i.e. sulfate and H_2_PO_4_ counterions), the large molecules are capable of significantly reducing the rate of aggregation at all concentrations. This stems from the behavior resulting from attractive guanidinium-anion interactions, which are lacking for the guanidinium chloride modified dendrimers. As indicated during molecular simulation snapshots, attractive guanidinium-sulfate/H_2_PO_4_ interactions cause dendrimer molecules to form clusters in solution and in return, inhibit multiple dendrimer arms from simultaneously binding to the protein, as indicated by radial distribution function plots. This reduced level of preferential binding producing a scenario in which the additive clusters solvate the surface of the protein, which reduces protein-protein interactions, without promoting unfolding. The elucidation of this particular type of additive gives insight into the behavior of PAMAM dendrimers in general, but more importantly, it demonstrates the role additive-additive interactions play in proteins stability.

## Materials and Methods

### Materials

Generation 0 through Generation 2 Dendritech® PAMAM Dendrimers with ethylenediamine cores, Bovine α-Chymotrypsinogen A type II (C4879), and jack bean Concanavalin A (C2010) were obtained from Sigma-Aldrich (St. Louis, MO). All other reagents were obtained from Sigma-Aldrich in the highest available grade. The concentration of aCgn and Con A were determined spectrophotometrically using extinction coefficient of 1.97 mL*mg^−1^ cm^−1^ at 282 nm and 1.37 mL*mg^−1^ cm^−1^ at 282 nm, respectively. All aCgn samples were pretreated with the enzymatic inhibitor phenylmethylsulfonyl fluoride and dialyzed against 20 mM sodium citrate, pH 5.

### Dendrimer Surface Modification

The PAMAM dendrimer surface amine groups were guanylated with an excess of 1,3-Bis(*tert*-butoxycarbonyl)-2-methyl-2-thiopseudourea in dimethylformamide (DMF). The DMF was evaporated and the residue dissolved in diethyl ether. The product was purified by repeated precipitation with n-hexane. The BOC protecting groups were removed with 4 M HCl dissolved in dioxane and the resulting salts were washed with acetone. For alternate salt forms, the counterions were exchanged using Amberlite IRA 400 anion exchange resin loaded using the appropriate sodium salt. The purity and structure were analyzed with NMR and mass spectrometry.

### Accelerated Aggregation

The aggregation of aCgn and Con A were accelerated by incubating samples at an elevated temperature in a Bio-Rad MyCycler thermal cycler. Aggregate formation and monomer loss was monitored using an Agilent 1200 series HPLC, equipped with a Zorbax GF-250 (4.6×250 mm, 4 micron) size exclusion column and a UV-Vis detector.

### Differential Scanning Calorimetry

The thermodynamic stability of 1 mg/mL solutions of aCgn in the presence of the modified dendrimers was determined by differential scanning calorimetry (Microcal VP-Differential Scanning Calorimeter) using a scan rate of 90°C/hour.

### Molecular Dynamics Simulations

Molecular dynamics (MD) simulations of aqueous solutions of the guanidinium modified generation 0 dendrimer salts with and without aCgn (PDB Id: 2CGA) were performed using NAMD 2.7 [32], with CHARMM27 [33] force fields and the TIP3P [34] water model. The force field parameters for the counterions were taken from the literature [35] and the force field parameters for the surface modified generation 0 dendrimer were developed using the CHARMM force field development procedure [36].

### Preferential Interaction Coefficient

Theoretical preferential interaction coefficient (Γ_23_) values were calculated using a statistical mechanical method applied to and all-atom model with no adjustable parameters [25]. Experimental values were obtained from changes in water activity as determined by vapor pressure osmometry [37]. Please see the Text S1 for complete details of all methods utilized.

## Supporting Information

Text S1 **Supporting Information Text.** Complete details of experimental and computational methods and results for NMR and mass spectroscopy analysis.
(DOCX)

Figure S1 **Convergence of preferential interaction coefficient (Γ_23_) of α-Chymotripsinogen A in aqueous dendrimer (GdmCl surface) salt solution.** The first 10 ns of instantaneous data are not used for calculation of cumulative averages.
(TIF)

Figure S2 **The influence of generation 0 PAMAM dendrimers, with surfaces modified to guanidinium, on aCgn monomer loss due to aggregation at 52.5°C.** The figure depicts aCgn monomer concentration, M, versus time relative to the initial monomer concentration, M_0_, of 10 mg/mL, with all solutions prepared in a 20 mM sodium citrate pH 5 buffer and all profiles fitted to a 2^nd^ order rate law. (A) Monomer loss profiles for solutions containing the guanidinium chloride salt form at varying concentrations. (B) Monomer loss profile for a solution containing the guanidinium dihydrogen phosphate salt form at a concentration of 0.2 M. The profiles for a solution containing no additive and a solution containing arginine hydrochloride at a concentration of 0.67 M are included for comparison.
(TIFF)

Figure S3 **Preferential Interaction Coefficient, Γ** ***_μ_***_**3**_ **, values versus additive concentration.** Values are for the interaction between generation 0 PAMAM dendrimers, with surfaces modified to guanidinium, and aCgn, as determined from VPO measurements. Error bars left off for clarity and curves drawn through the plots to aid the eye (see Table S2 for more detail).
(TIF)

Figure S4 **Snapshots of PAMAM dendrimer and counter-ions within 0.6 nm of the protein surface.** Guanidinium chloride surface (left) and Guanidinium sulfate surface (right).
(TIF)

Table S1 **Setup of simulation systems.**
(DOCX)

Table S2 **Summary of additive molecular weight, partial molar volume (V), preferential interactions with aCgn as determined by VPO, and aCgn denaturation midpoint temperature increment as determined by DSC for surface modified PAMAM dendrimers.**
(DOCX)
