# Supplementary material for: Effects of Solute-Solute Interactions on Protein Stability Studied Using Various Counterions and Dendrimers
Source: PLoS One. 2011 Nov 18;6(11):e27665. doi: 10.1371/journal.pone.0027665 (PMC3220676; doi:10.1371/journal.pone.0027665)
Supplement: Text S1 — Supporting Information Text. Complete details of experimental and computational methods and results for NMR and mass spectroscopy analysis. (DOCX) [file pone.0027665.s001.docx]

Supporting Information for:

**Effects of Solute-Solute Interactions on Protein Stability Studied Using Various Counterions and Dendrimers**

Curtiss P. Schneider^1^, Diwakar Shukla^1^, and Bernhardt L. Trout^2^

*Department of Chemical Engineering, Massachusetts Institute of Technology, 77 Massachusetts Avenue, Cambridge, Massachusetts 02139*

^1^These authors contributed equally to this work.

^2^To whom correspondence should be addressed.

77 Massachusetts Avenue

E19-502b

Cambridge, MA 02139

Tel: (617) 258-5021

Fax: (617) 253-2272

Email: trout@mit.edu

**Experimental Methods**

**Materials.** Generation 0 through Generation 2 Dendritech® PAMAM Dendrimers with ethylenediamine cores, Bovine α-chymotrypsinogen A type II, aCgn, (C4879) and jack bean Concanavalin A, Con A, (C2010) were obtained from Sigma-Aldrich (St. Louis, MO). 1,3-Bis(*tert*-butoxycarbonyl)-2-methyl-2-thiopseudourea, dimethylformamide, diethyl ether, 4M hydrogen chloride dissolved in dioxane, n-hexane, acetone, dimethyl sulfoxide-d6, sodium phosphate monobasic, phosphoric acid (85%), sodium sulfate, 5N sulfuric acid, sodium acetate, acetic acid, sodium chloride, 4M hydrochloric acid, citric acid monohydrate, sodium hydroxide, phenylmethylsulfonyl fluoride, and anhydrous ethanol were also obtained from Sigma-Aldrich in the highest available grade. The concentration of aCgn and Con A were determined spectrophotometrically with a PerkinElmer Lambda 35 UV/Vis spectrometer using extinction coefficient of 1.97 mL*mg^-1^cm^-1^ at 282 nm and 1.37 mL*mg^-1^cm^-1^ at 282 nm, respectively [1].

**Surface Modification.** Dendritech® PAMAM dendrimers (generations 0-2) containing ethylenediamine cores were guanylated with an excess of 1,3-Bis(*tert*-butoxycarbonyl)-2-methyl-2-thiopseudourea in dimethylformamide (DMF), with the reaction monitored via TLC. After the reaction was completed (typically within 48-72 hours), the majority of the DMF was evaporated at 60^o^C under reduced pressure and the excess guanylating agent was removed by dissolving the residue in a minimal amount of diethyl ether and precipitating the product with hexane. After discarding the supernatant, the precipitates were redissolved in diethyl ether and the procedure was repeated until the product was pure (as indicated by NMR). The BOC protecting group was removed from the purified product with 4M HCl dissolved in dioxane, producing a PAMAM dendrimer with a surface modified to guanidinium chloride, which precipitated from the solution. The precipitated product was washed four times with diethyl ether, followed by four washes with acetone, and then dried at an elevated temperature and reduced pressure. The structures of the modified dendrimers were verified via NMR and mass spectrometry.

Ion exchange was utilized for preparing the phosphate, sulfate, and acetate salt forms of the modified dendrimers. An excess of sodium acetate, sodium phosphate, monobasic or sodium sulfate was utilized to load the anion desired onto a column containing Amberlite IRA 400 anion exchange resin. After rinsing the column with HPLC grade water, a solution containing a modified dendrimer was passed through the column, in an amount less than half of the capacity of the column and at the recommended flowrate to ensure complete ion exchange. Additional water was then passed through the column to elute all of the excipient. Excess water was evaporated at 60^o^C under reduced pressure with a rotavap and the product was dried to completeness via lyophilization.

**Accelerated Aggregation.** The aggregation of α-Chymotrypsinogen A (aCgn) and Concanavalin A (Con A) were accelerated by incubating samples at an elevated temperature. Most experiments used aCgn as the model protein, which was selected because it is a well characterized protein that has been used extensively as a model protein in aggregation studies due to it having aggregation characteristics similar to therapeutically relevant proteins [2]. Con A was selected as a model diagnostic protein. The temperature range utilized varied from 37 to 52.5^o^C. In addition to all aggregation experiments being conducted at elevated temperatures, all samples contained 10 mg/mL aCgn, held at pH 5.0 using a 20 mM sodium citrate buffer or 7.5 mg/mL Con A, held at pH 6.5 using 40 mM sodium phosphate buffer. 50 μL aliquots of each protein-cosolute mixture were placed in 0.2 mL PCR tubes and then incubated in a Bio-Rad MyCycler thermal cycler with a timer initiated when the thermal cycler reached the desired temperature. Each series contained 12 samples and up to 8 different mixtures were tested at the same time during any given experiment. Samples were removed periodically and immediately placed in an ice bath to quench the aggregation reaction. The length of each experiment was planned so that the extent of the reaction was such that 5-20% of the protein aggregated, making changes in the rate of aggregation pronounced. After a minimum of five minutes in the ice bath, samples were briefly spun in a minicentrifuge to remove precipitated aggregates and then transferred to autosampler vials. 15 μL of each sample were injected into an Agilent 1200 series HPLC, equipped with a Zorbax GF-250 (4.6x250 mm, 4 micron) size exclusion column and a UV-Vis detector, during a sequence. The HPLC mobile phase for aCgn analysis contained 25 mM citric acid monohydrate, 25 mM sodium acetate, and 200 mM sodium chloride with the pH adjusted to 4.0 using sodium hydroxide and the HPLC mobile phase for Con A analysis contained 50 mM sodium phosphate, monobasic and 200 mM sodium chloride with the pH adjusted to 6.5 using sodium hydroxide. The flowrate was set at 1 mL/min and samples were monitored at 280 nm.

Most of the aggregates that formed either precipitated or did not pass through the column, thus only the monomer concentration was monitored with time. Peak area at each time point was compared to the peak area for an unstressed sample to create a monomer loss profile normalized with respect to the initial concentration. Each profile was compared to a profile for a sample containing no cosolute to determine the change in aggregation rate upon addition of the cosolute. For a control, each aggregation experimental setup had one protein solution that contained no cosolute to verify that the baseline aggregation rate remained constant amongst all experiments. For all aCgn concentrations studied, the monomer loss rate exhibited 2^nd^ order kinetics, thus rate constants were determined by fitting each data set to a 2^nd^ order rate law. Con A aggregation exhibited higher order kinetics and comparisons were made by analyzing the amount of time for a 5% loss of the monomeric protein. It should also be noted that the sample of aCgn attained contained trace amounts of chymotrypsin or trypsin, which exhibits proteolytic activity at pH>4.5. All samples were first treated with the enzymatic inhibitor phenylmethylsulfonyl fluoride (PMSF).

**Enzymatic Inhibition and aCgn Solution Preparation.** When incubated at elevated temperatures, the digestion rate of aCgn is comparable to the rate of aggregation, leading to a considerable amount of small molecular weight peptides forming over the course of an experiment and a considerable amount of the protein lost to digestion rather than aggregation. Lowering the pH was not an option because the stability of the protein is reduced from being in such a highly charged state (aCgn has a pI of 9.1) and is highly sensitive to the presence of ions, which screen the repulsive electrostatic interactions. A pH around 5 is optimal for when using this protein as a model system because the protein is least sensitive to the presence of ions at that pH [3]. To verify this, sodium chloride and potassium chloride were used as cosolutes, up to a concentration of 1M, which exhibited no influence on the rate of aggregation. To keep the protein from being digested, a stock of aCgn was first treated with phenylmethylsulfonyl fluoride (PMSF), a serine protease inhibitor that irreversibly inhibits proteolytic activity. 35 mg of PMSF was first dissolved in 1 mL of anhydrous ethanol. This solution was then added in 250 μL increments to a stirred 50 mL stock solution of aCgn (10 mg/mL, 100 mM sodium phosphate pH 7) every hour until PMSF reached its solubility limit, usually after three additions. Adding PMSF in increments was utilized because the inhibitor, though effective at low concentrations, rapidly degrades in aqueous solutions [4]. Three hours after the first addition of PMSF, the solution was passed through a 0.1 micron filter (Millipore) to remove PMSF precipitates, then placed under dialysis (Spectra/Por 3500 MWCO) for 48 hours at 4^o^C against three changes of 20 mM sodium citrate pH 5 buffer (700 mL each). Following the dialysis, the solution was first centrifuged briefly to remove any aggregates that precipitated during dialysis and then concentrated using a centrifugal filter (Amicon Ultra 10,000 MWCO) to a final concentration of 20 mg/mL. This treatment reduced the digestion rate to an insignificant level and all aCgn samples produced in this manner had near identical aggregation rates. All aCgn solutions were frozen, rather than lyophilized, in 0.3 mL aliquots and used within 6 months. Storing the protein in this manner did not seem to have any detrimental effect on the quality of the protein (i.e. the amount of aggregates in the initial solution) nor the rate of aggregation when used in the study. Solutions for the aggregation study were prepared by first preparing a solution of the cosolute of interest in a 20 mM sodium citrate pH 5 buffer at a concentration double that of interest (adjusting the pH to 5 if needed with the appropriate acid or an 8M sodium hydroxide solution), then mixing 0.3 mL of this solution with 0.3 mL of a thawed aCgn solution. This solution was then divided into twelve aliquots and used as described in the main article.

**Differential Scanning Calorimetry.** The thermodynamic stability of aCgn in the presence of the modified dendrimers was determined by differential scanning calorimetry (DSC). A Microcal VP-Differential Scanning Calorimeter was utilized and each reading began with a minimum of three buffer-buffer up and down scans (in this case, the buffer also contains the cosolute of interest) to establish a reproducible thermal history followed by a single protein-buffer up scan. aCgn was analyzed at a concentration of 1 mg/mL in a 20 mM sodium citrate pH 5 buffer containing the cosolute of interest and a scan rate of 90^o^C/hour. The data was analyzed in the MicroCal Origin® plotting software. In the analysis, the last buffer-buffer reference scan was first subtracted from the data and then the data was normalized with respect to the protein concentration, cell volume, and scan rate. The baseline (which was progressed through the peak) was then subtracted from the plot and the denaturation midpoint temperature, T_m_, was taken as the temperature at the peak of the unfolding event. For each dendrimer salt form, three concentrations (including zero concentration) were tested and T_m_ values with respect to cosolute concentration were fitted to a linear trend.

**VPO Preferential Interaction Methodology.** Preferential interaction theory is the thermodynamic framework to quantify the effect of cosolutes on protein stability. The preferential interaction coefficient, Γ*_μ_*_3_, is a measure of the preference of cosolutes for the protein surface and is defined by the following expression,

$\Gamma_{\mu_{3}}\equiv\left(\frac{\partial m_{3}}{\partial m_{2}}\right)_{T,P,\mu_{3}}=-\left(\frac{\partial\mu_{2}}{\partial\mu_{3}}\right)_{T,P,m_{2}}$,

where *m, T, P and μ* represent molal concentration, temperature, pressure, and chemical potential, respectively.[5] The subscripts used indicate solution components in Scatchard notation: water (subscript 1), the protein (subscript 2), and the cosolute (subscript 3).[6] Additives with a positive Γ*_μ_*_3_ are typically described as being preferentially bound to the protein surface due to an increase in the concentration of the cosolute in the local domain and this favorable interaction, as indicated by eq 1, lowers the chemical potential of the protein. The opposite is true for additives with a negative Γ*_μ_*_3_, which are typically described as being preferentially excluded from the surface of the protein. Preferential interactions, though weak in nature, will significantly influence the solubility and stability of a protein in addition to influencing protein-protein interactions [7].

We have previously used VPO to determine the preferential interaction coefficient of ArgHCl.[8] In that article, we thoroughly explain the implications of this parameter and how to obtain values utilizing the VPO methodology, thus complete details of the procedure will not be repeated here. In this report, a Wescor Vapro® 5520 vapor pressure osmometer was utilized to determine the osmolality of three separate series (16 samples in each series) of carefully prepared solutions (i.e. cosolute only solutions, protein only solutions, and protein-cosolute solutions at a constant protein concentration), the goal being to determine the slope with respect to concentration. For each series, 16 samples containing 80 μL of solution of varying concentration were prepared, each with a constant concentration of buffer. Stock solutions were prepared first and individual samples were prepared gravimetrically from these solutions using a Mettler Toledo balance with a precision of 0.1 mg. The first stock solution contained only the cosolute and water (pH adjusted to 5) and the second stock solution contained 100 mg/mL of aCgn in a 40 mM sodium citrate pH 5 buffer, prepared in a fashion similar to the stock solution used in the aggregation experiments. The cosolute only series was prepared by diluting a varying amount of the stock solution with water to produce a 40 μL solution and then adding 40 μL of the 40 mM sodium citrate pH 5 buffer. The protein-cosolute solution was prepared in a similar fashion but with 40 μL of the protein stock solution added to each solution instead of buffer. The protein only solution was prepared by simply diluting the protein stock solution with buffer.

The osmometry readings were replicated in triplicate, with the osmometer recalibrated after every four readings using 0.1, 0.29, and 1 mol/kg osmolality standards and the thermocouple cleaned daily with concentrated ammonium hydroxide and a rinse with HPLC grade water. The trend with respect to concentration was fitted to a 2^nd^ order polynomial. As described before, osmolality readings were kept below 1.2 mol/kg for improved accuracy [8]. The preferential interaction coefficient reported was determined by averaging the values obtained from the two different methods for approximating the parameter from osmometry data:

$\Gamma_{\mu_{3}}=\frac{1}{2}\left(\Gamma_{\mu_{3}}^{I}+\Gamma_{\mu_{3}}^{II}\right)$.

The equations obtained from those two approaches can be expressed by

$$\Gamma_{\mu_{3}}^{I}\cong\frac{m_{3}}{m_{2}}\left[1-\frac{\Omega_{3}}{\Omega_{3}^{\circ(2)}}\right]$$

and

$$\Gamma_{\mu_{3}}^{II}\cong\left[\frac{m_{3}\left(\Omega_{2}^{\circ(3)}+\Gamma_{\mu_{1}}\Omega_{3}\right)}{m_{3}\Omega_{3}+m_{2}\left(\Omega_{2}^{\circ(3)}+\Gamma_{\mu_{1}}\Omega_{3}\right)}\right]$$

with the additional parameters expressed by

$$\Omega_{k}=\left(\frac{\partial Osm}{\partial m_{k}}\right)_{T,P,m_{k'\neq k}}$$

and

$\Gamma_{\mu_{1}}=\left(\frac{\partial m_{3}}{\partial m_{2}}\right)_{T,P,\mu_{1}}\cong\frac{\left(m_{3}-m_{3}^{\Delta}\right)}{m_{2}}$.

In the above equations, the superscript o(*i*) represents a two component solution lacking component *i*, *Osm* represents osmolality and *m*_3_^Δ^ represents the molal concentration of a cosolute only solution at the same osmolality (i.e. the same water chemical potential) as a protein-cosolute solution.

**NMR Spectrometry.** A Bruker Avance 400 NMR spectrometer was utilized to analyze the structure and purity of the modified dendrimers. 1D ^1^H (400 MHz) and 1D ^13^C (101 MHz) spectrums were produced for official validation of the structure. 2D ^1^H-^1^H COSY and 2D ^1^H-^13^C HSQC techniques were utilized to help deconvolute the 1D spectrums. If any ambiguity persisted, results were compared to predictions made using ChemBioDraw Ultra 12.0. The guanylating agent and amine precursors were analyzed as well (which also included the amine in an ammonium chloride salt form for comparison to the guanidinium salt spectrum) to identify any unreacted precursors. The amine starting compounds and the BOC protected intermediates were each dissolved in deuterated chloroform (CDCl_3_) and the guanidinium salt products and ammonium salt precursors were dissolved in deuterated dimethyl sulfoxide (DMSO-d6), all at a concentration around 25 mg/mL. ^1^H and ^13^C spectrums were calibrated using residual protio-solvent. The total integrated area of the ^1^H spectrums were set to the total number of hydrogens present on the molecule. No unreacted amine or guanylating agent were identified, thus the only impurities were residual solvent, which were identified by comparing chemical shift values with literature values.

**Mass Spectrometry.** Electrospray ionization (ESI) was deemed best suited for analyzing the modified dendrimer compounds with minimal fragmentation. A Bruker Daltonics APEXIV 4.7 Tesla Fourier Transform Ion Cyclotron Resonance Mass Spectrometer (FT-ICR-MS) equipped with an electrospray ionization source was utilized. Only positive ions were analyzed and the data was deemed satisfactory when the major peaks corresponded to [M+nH]^n+^ values.

**Computational Methods**

**Simulation setup.** Molecular dynamics (MD) simulations of aqueous dendrimer (generation 0) salt solutions were performed using NAMD 2.7 [9], with CHARMM27 [10] force fields and the TIP3P [11] water model. The force field parameters for counterions were taken from the literature [12]. The force-field parameters for surface modified generation 0 dendrimer were developed using the CHARMM force field development procedure [13]. The partial charges and parameters for the arms of the dendrimers with guanidinium terminal groups were taken from the force field parameters for arginine. Due to the unavailability of the experimental data related to these compounds, data obtained from quantum calculations was used to validate the force field. For the adjustment of the partial atomic charges, the QM interactions with water be calculated at the HF/6-31G* level of theory to ensure compatibility with the current CHARMM force fields. For the purpose of force field development, the dendrimer molecule was decomposed into molecular fragments, with ethylene diamine core as one fragment and the dendrimer arm as another fragment. Periodic boundary conditions were applied with long-range electrostatic interactions beyond the non-bonded cutoff of 10 Å accounted for using the Particle Mesh Ewald (PME) method [14] with the grid spacing on the order of 1 Å or less. Pressure was maintained at 1 atm using the Langevin piston method, with a piston period of 200 fs, a damping time constant of 100 fs, and a piston temperature of 298 K [15]. The initial size of the periodic rectangular box containing dendrimer, counter-ion and water was set to (50 Å)^3^ in all simulations. To set up the simulation systems for various dendrimer salts, dendrimer molecules and counter-ions were placed randomly (without overlap) in the box, and subsequently the overlapping water molecules were removed such that the molality of the solution is set to 0.2. The total simulation time for each run was 100 ns, with a time step of 1 fs and sample collection every 1 ps.

MD simulations of α-Chymotrypsinogen A (PDB Id: 2CGA) were also performed in presence of aqueous dendrimer salt solutions. The initial size of the periodic rectangular box containing protein and water was set such that there was at least a 15 Å thick layer of water around the protein in each direction. The total simulation time for each run was 100 ns, with a time step of 1 fs.

The details of the simulation setup are included in Table S1. A total of 600 ns of simulations were performed for these systems. The simulated trajectories were analyzed mainly in terms of radial distribution functions and number of hydrogen bonds between protein, dendrimer and counter-ions. Theoretical preferential interaction coefficients are also calculated to assess the validity of the developed force field and to understand the nature of interactions between protein and dendrimer salts. The extent of clustering in dendrimer salts solutions was quantified in terms of the loss of solvent accessible surface area of dendrimer molecules. The procedures used for estimating these quantities are included below.

**Preferential Interaction Coefficient.** The procedure for calculating the preferential interaction coefficient (Γ_23_) based on a statistical mechanical method applied to all-atom model with no adjustable parameters was used [16]. The number of dendrimer, counterion and water molecules within a bin of 0.1 Å as a function of distance from the protein surface is used to calculate Γ_23_. The expression used to calculate the coefficient is [17,18]:

$\Gamma_{23}(t)=\left\langle n_{3}^{local}-\frac{n_{3}^{bulk}}{n_{1}^{bulk}}n_{1}^{local}\right\rangle$. (7)

In the above equation, *n* denotes the number of a specific type of molecule (subscript 3 for the cosolute, subscript 2 for the protein, and subscript 1 for water) in a certain domain (superscript *bulk* for a bulk volume outside of the vicinity of the protein and superscript *local* for a volume in the protein vicinity), and angle brackets denote an ensemble average. The extent of the local domain was chosen to be 6 Å. For electrolyte solutions, preferential interaction coefficient for both the cation and anion were evaluated separately and then averaged using

$\Gamma_{23}=0.5\left(\Gamma_{2,+3}+\frac{\nu_{+}}{\nu_{-}}\Gamma_{2,-3}\right)$ (8)

where $\Gamma_{2,+3}$ is the preferential interaction for cation, $\Gamma_{2,-3}$ is the preferential interaction coefficient for anion, $\nu_{+}$and $\nu_{-}$ are the number of molecules of cation and anion in the chemical formula of the salt. The net charge of the protein is subtracted from the preferential interaction coefficient value for anion or cation based on whether the protein is positively or negatively charged respectively.

The preferential interaction coefficient values converge within 100 ns (see Figure S1). However, due to the formation of clusters, the diffusivity of individual dendrimer molecules is significantly reduced for sulfate and dihydrogen phosphate salts. Therefore, it is expected that the preferential interaction coefficient values for these salts could change with simulation time.

**Hydrogen bonds.** The number of hydrogen bonds was calculated using the standard CHARMM tools with a cutoff radius of 2.5 Å between hydrogen atom and acceptor atom and a cutoff angle of 30°.

**Clustering.** Self-aggregation or clustering of dendrimer molecules was quantified in terms of the reduction of total solvent accessible surface area. The solvent accessible area was estimated using standard CHARMM commands with a probe sphere of 1.4 Å radius. The solvent accessible surface area was used as a measure, since minimization of exposed surface area is one of the main consequences of clustering.

**Purity, Yield, NMR, and MS Data for Guanidine-Modified PAMAM Dendrimers**

**BOC Protected Guanidine Modified PAMAM Dendrimers**

**Generation 0 (G0-BOC)**

[^a^CH_2_^a^CH_2_]-(^b^N[^c^CH_2_^d^CH_2_^e^CO^f^NH^g^CH_2_^h^CH_2_^i^NH^j^C(^k^NH^l^COO^m^C(^n^CH_3_)_3_)(^k’^N^l’^COO^m’^C(^n’^CH_3_)_3_)]_2_)_2_

Purity (NMR): 91.4 %

^1^H-NMR (400 MHz, CDCl_3_): δ ppm 11.40 (4H, s, k), 8.51 (4H, t, J=5.5 Hz, i), 7.94 (4H, t, J=4.7 Hz, f), 3.50 (8H, q, J=5.7 Hz, h), 3.34 (8H, q, J=5.6 Hz, g), 2.61 (8H, t, J=5.5 Hz, c), 2.35 (4H, s, a), 2.29 (8H, t, J=5.6 Hz, d) , 1.433 (36H, s, n’), 1.439 (36H, s, n)

^13^C-NMR (101 MHz, CDCl_3_): δ ppm 173.3 (e), 163.4 (j), 157.0 (l’), 153.1 (l), 83.4 (m), 79.5 (m’), 52.2 (a), 50.5 (c), 40.6 (h), 39.8 (g), 34.3 (d), 28.2 (n’), 28.5 (n)

MS (ESI^+^): m/z calculated for C_66_H_120_N_18_O_20_ [M+H]^+^: 1485.90; found: 1485.91 (100%): [M+2H-COOC(CH_3_)_3_]^+^: 1385.85; found: 1385.86 (43%), [M+Na]^+^: 1507.88; found: 1507.89 (38%).

**Generation 1 (G1-BOC)**

[^a^CH_2_^a^CH_2_]-[^b1^N(^c1^CH_2_^d1^CH_2_^e1^CO^f1^NH^g1^CH_2_^h1^CH_2_^b2^N[^c2^CH_2_^d2^CH_2_^e2^CO^f2^NH^g2^CH_2_^h2^CH_2_^i^NH^j^C(^k^NH^l^COO^m^C(^n^CH_3_)_3_)(^k’^N^l’^COO^m’^C(^n’^CH_3_)_3_)]_2_)_2_]_2_

Purity (NMR): 90.6 %

^1^H-NMR (400 MHz, CDCl_3_): δ ppm 11.37 (8H, s, k), 8.51 (8H, t, J=5.5 Hz, i), 7.95 (8H, t, J=5.3 Hz, f2), 7.69 (4H, t, J=5.4 Hz, f1), 3.49 (16H, q, J=5.6 Hz, h2), 3.33 (16H, q, J=5.4 Hz, g2), 3.18 (8H, q, J=5.7 Hz, g1), 2.65-2.72 (24H, br t, c1+c2), 2.49 (8H, t, J=5.7 Hz, h1), 2.45 (4H, s, a), 2.28-2.31 (24H, br t, d1+d2) , 1.43 (72H, s, n’), 1.42 (72H, s, n)

^13^C-NMR (101 MHz, CDCl_3_): δ ppm 172.8-173.0 (e1+e2), 163.3 (j), 157.0 (l’), 153.1 (l), 83.4 (m), 79.4 (m’), 52.3-52.5 (a+h1), 50.4-50.5 (c1+c2), 40.6 (h2), 40.0 (g2), 37.6 (g1), 34.1-34.2 (d1+d2), 28.2 (n’), 28.5 (n)

MS (ESI^+^): m/z calculated for C_150_H_272_N_42_O_44_ [M+2H]^2+^: 1684.02; found: 1684.54 (100%), [M+3H-COOC(CH_3_)_3_]^3+^: 1633.99; found: 1634.52 (97%), [M+Na]^+^: 1695.51; found: 1695.53 (53%).

**Generation 2 (G2-BOC)**

[^a^CH_2_^a^CH_2_]-(^b1^N[^c1^CH_2_^d1^CH_2_^e1^CO^f1^NH^g1^CH_2_^h1^CH_2_^b2^N(^c2^CH_2_^d2^CH_2_^e2^CO^f2^NH^g2^CH_2_^h2^CH_2_ ^b3^N[^c3^CH_2_^d3^CH_2_^e3^CO^f3^NH^g3^CH_2_^h3^CH_2_ ^i^NH^j^C(^k^NH^l^COO^m^C(^n^CH_3_)_3_)(^k’^N^l’^COO^m’^C(^n’^CH_3_)_3_)]_2_)_2_]_2_)_2_

Purity (NMR): 94.9 %

^1^H-NMR (400 MHz, CDCl_3_): δ ppm 11.37 (16H, s, k), 8.51 (16H, t, poorly resolved, i), 7.96 (16H, t, poorly resolved, f3), 7.82 (4H, t, poorly resolved, f1), 7.64 (8H, t, poorly resolved, f2), 3.49 (32H, q, J=4.9 Hz, h3), 3.33 (32H, q, J=4.3 Hz, g3), 3.18-3.21 (24H, br t, g1+g2), 2.65-2.72 (56H, br t, c1+c2+c3), 2.45-2.49 (28H, br m, a+h1+h2), 2.25-2.32 (56H, br t, d1+d2+d3) , 1.424 (144H, s, n’), 1.418 (144H, s, n)

^13^C-NMR (101 MHz, CDCl_3_): δ ppm 172.9-173.1 (e1+e2+e3), 163.3 (j), 157.0 (l’), 153.0 (l), 83.3 (m), 79.4 (m’), 52.4-52.6 (h1), 50.3-50.4 (c1+c2+c3), 40.6 (h3), 40.0 (g3), 37.5-37.6 (g1+g2), 34.1-34.2 (d1+d2+d3), 28.2 (n’), 28.5 (n)

MS (ESI^+^): m/z calculated for C_318_H_576_N_90_O_92_ [M+20H]^20+^: 357.42; found: 357.22 (100%)

**Guanidine HCl Modified PAMAM Dendrimers**

**Generation 0 (G0-Gdn)**

[^a^CH_2_^a^CH_2_]-(^b^NH[^c^CH_2_^d^CH_2_^e^CO^f^NH^g^CH_2_^h^CH_2_^i^NH^j^C(^k^NH_2_)_2_]_2_)_2_

Purity (NMR): 77.0 %

Yield: 81.8 %

^1^H-NMR (400 MHz, DMSO-d_6_): δ ppm 11.06 (2H, br s, b), 8.50 (4H, t, J=5.1 Hz, i), 7.89 (4H, t, J=5.2 Hz, f), 7.34 (16H, br s, k), 3.72 (4H, br s, a), 3.43 (8H, t, J=6.3 Hz, c), 3.20-3.24 (16H, br m, g+h), 2.77 (8H, t, J=6.9 Hz, d)

^13^C-NMR (101 MHz, DMSO-d_6_): δ ppm 169.3 (e), 157.3 (j), 49.2 (c), 46.4 (a), 40.1 (g), 38.0 (h), 29.3 (d)

MS (ESI^+^): m/z calculated for C_26_H_62_N_18_O_4_Cl_6_ [M-6HCl+2H]^2+^: 343.24; found: 343.25 (100%); [M-6HCl+3H]^3+^: 229.17; found: 229.17 (59%), [M-6HCl+4H]^4+^: 172.13; found: 172.13 (16%), [M-6HCl+H]^+^: 685.48; found: 685.51 (4%).

**Generation 1 (G1-Gdn)**

[^a^CH_2_^a^CH_2_]-[^b1^NH(^c1^CH_2_^d1^CH_2_^e1^CO^f1^NH^g1^CH_2_^h1^CH_2_^b2^NH[^c2^CH_2_^d2^CH_2_^e2^CO^f2^NH^g2^CH_2_^h2^CH_2_^i^NH^j^C(^k^NH_2_)_2_]_2_)_2_]_2_

Purity (NMR): 70.4 %

Yield: 78.5 %

^1^H-NMR (400 MHz, DMSO-d_6_): δ ppm 11.09 (2H, br s, b1), 10.45 (4H, br s, b2), 8.67 (4H, t, poorly resolved, f1), 8.51 (8H, t, poorly resolved, i), 7.91 (8H, t, poorly resolved, f2), 7.36 (32H, br s, k), 3.73 (4H, br s, a), 3.45-3.53 (8H, br q, g1), 3.35-3.45 (24H, br t, c1+c2), 3.18-3.27 (40H, br m, h1+h2+g2), 2.71-2.85 (24H, br t, d1+d2)

^13^C-NMR (101 MHz, DMSO-d_6_): δ ppm 169.5-169.6 (e1+e2), 157.3 (j), 51.3 (h1), 48.7-48.8 (c1+c2), 46.5 (a), 40.2 (g2), 38.1 (h2), 33.7 (g1), 29.1-29.3 (d1+d2)

MS (ESI^+^): m/z calculated for C_70_H_158_N_42_O_12_Cl_14_ [M-14HCl+6H]^6+^: 295.21; found: 295.21 (100%), [M-14HCl+5H]^5+^: 354.05; found: 354.06 (90%), [M-14HCl+4H]^4+^: 442.31; found: 442.32 (70%), [M-14HCl+7H]^7+^: 253.18; found: 253.18 (62%), [M-14HCl+3H]^3+^: 589.41; found: 589.43 (27%).

**Generation 2 (G2-Gdn)**

[^a^CH_2_^a^CH_2_]-(^b1^NH[^c1^CH_2_^d1^CH_2_^e1^CO^f1^NH^g1^CH_2_^h1^CH_2_^b2^NH(^c2^CH_2_^d2^CH_2_^e2^CO^f2^NH^g2^CH_2_^h2^CH_2_ ^b3^NH[^c3^CH_2_^d3^CH_2_^e3^CO^f3^NH^g3^CH_2_^h3^CH_2_^i^NH^j^C(^k^NH_2_)_2_]_2_)_2_]_2_)_2_

Purity: 77.7 %

Yield: 81.1 %

^1^H-NMR (400 MHz, DMSO-d_6_): δ ppm 11.09 (2H, br s, b1), 10.40-10.44 (12H, br s, b2+b3), 8.62-8.69 (12H, br t, f1+f2), 8.51 (16H, t, poorly resolved, i), 7.91 (16H, br t, poorly resolved, f3), 7.34 (64H, br s, k), 3.65-3.75 (20H, br m, a+h2), 3.48-3.53 (24H, br q, g1+g2), 3.35-3.48 (56H, br t, c1+c2+c3), 3.18-3.27 (72H, br m, h1+h3+g3), 2.71-2.82 (56H, br t, d1+d2+d3)

^13^C-NMR (101 MHz, DMSO-d_6_): δ ppm 169.5-169.7 (e1+e2+e3), 157.3 (j), 51.3-51.4 (h1+h2), 48.7-48.8 (c1+c2+c3), 46.6 (a), 40.2 (g3), 38.1 (h3), 33.7-33.8 (g1+g2), 29.1-29.3 (d1+d2+d3)

MS (ESI^+^): m/z calculated for C_158_H_350_N_90_O_28_Cl_30_ [M-29HCl+11H]^11+^: 361.25; found: 361.26 (100%); [M-30HCl+8H]^8+^: 492.11; found: 492.11 (97%), [M-30HCl+9H]^9+^: 437.54; found: 437.54 (92%), [M-30HCl+10H]^10+^: 393.89; found: 393.88 (47%), [M-30HCl+7H]^7+^: 562.27; found: 562.27 (33%), [M-30HCl+6H]^6+^: 655.81; found: 655.81 (10%).

**References**

1. Gekko K, Timasheff SN (1981) Mechanism of Protein Stabilization by Glycerol: Preferential Hydration in Glycerol-Water Mixtures. Biochemistry 20: 4667-4676.

2. Andrews JM, Roberts CJ (2007) Non-Native Aggregation of α-Chymotrypsinogen Occurs through Nucleation and Growth with Competing Nucleus Sizes and Negative Activation Energies. Biochemistry 46: 7558-7571.

3. Velev OD, Kaler EW, Lenhoff AM (1998) Protein interactions in solution characterized by light and neutron scattering: Comparison of lysozyme and chymotrypsinogen. Biophys J 75: 2682-2697.

4. Gold AM, Fahrney D (1964) Sulfonyl Fluorides As Inhibitors Of Esterases .2. Formation + Reactions Of Phenylmethanesulfonyl Alpha-Chymotrypsin. Biochemistry 3: 783-&.

5. Courtenay ES, Capp MW, Anderson CF, Jr. MTR (2000) Vapor Pressure Osmometry Studies of Osmolyte-Protein Interactions: Implications for the Action of Osmoprotectants in Vivo and for the Interpretation of "Osmotic Stress" Experiments in Vitro. Biochemistry 39: 4455-4471.

6. Scatchard G (1946) Physical chemistry of protein solutions. I. Derivation of the equations for the osmotic pressure. J Am Chem Soc 68: 2315-2319.

7. Timasheff SN (1993) The Control of Protein Stability and Association by Weak Interactions With Water: How Do Solvents Affect These Processes? Annu Rev Biophys Biomolec Struct 22: 67-97.

8. Schneider CP, Trout BL (2009) Investigation of Cosolute-Protein Preferential Interaction Coefficients: New Insight into the Mechanism by Which Arginine Inhibits Aggregation. J Phys Chem B 113: 2050-2058.

9. Phillips JC, Braun R, Wang W, Gumbart J, Tajkhorshid E, et al. (2005) Scalable molecular dynamics with NAMD. J Comput Chem 26: 1781-1802.

10. Brooks BR, Bruccoleri RE, Olafson BD, States DJ, Swaminathan S, et al. (1983) Charmm - A Program For Macromolecular Energy, Minimization, And Dynamics Calculations. J Comput Chem 4: 187-217.

11. Jorgensen WL, Chandrasekhar J, Madura JD, Impey RW, Klein ML (1983) Comparison Of Simple Potential Functions For Simulating Liquid Water. J Chem Phys 79: 926-935.

12. Cannon WR, Pettitt BM, McCammon JA (1994) Sulfate Anion In Water - Model Structural, Thermodynamic, And Dynamic Properties. J Phys Chem 98: 6225-6230.

13. MacKerell AD (2001) Atomistic Models and Force Fields. In: Becker O, MacKerell AD, Roux B, Watanabe M, editors. Computation Biochemistry and Biophysics. New York: Marcel Dekker Inc. pp. 7-38.

14. Darden T, York D, Pedersen L (1993) Particle Mesh Ewald - An N.Log(N) Method For Ewald Sums In Large Systems. J Chem Phys 98: 10089-10092.

15. Feller SE, Zhang YH, Pastor RW, Brooks BR (1995) Constant-Pressure Molecular-Dynamics Simulation - The Langevin Piston Method. J Chem Phys 103: 4613-4621.

16. Baynes BM, Trout BL (2003) Proteins in mixed solvents: A molecular-level perspective. J Phys Chem B 107: 14058-14067.

17. Shukla D, Shinde C, Trout BL (2009) Molecular Computations of Preferential Interaction Coefficients of Proteins. J Phys Chem B 113: 12546-12554.

18. Shukla D, Trout BL (2010) Interaction of Arginine with Proteins and the Mechanism by Which It Inhibits Aggregation. J Phys Chem B 114: 13426-13438.
