# Supplementary figures and images for: Effects of Solute-Solute Interactions on Protein Stability Studied Using Various Counterions and Dendrimers
Source: PLoS One. 2011 Nov 18;6(11):e27665. doi: 10.1371/journal.pone.0027665 (PMC3220676; doi:10.1371/journal.pone.0027665)

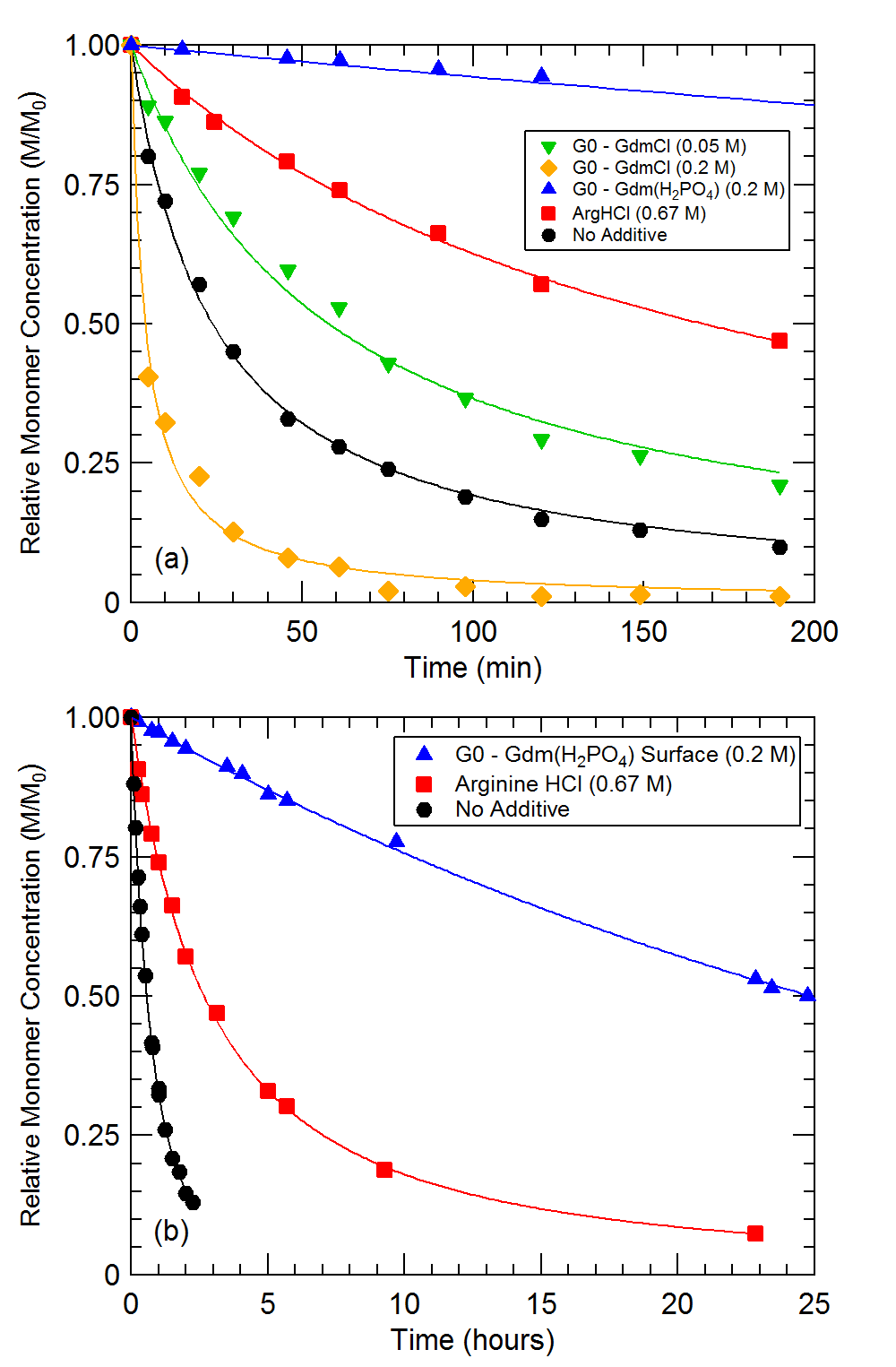

Supplement: Figure S1 — Convergence of preferential interaction coefficient (Γ23) of α-Chymotripsinogen A in aqueous dendrimer (GdmCl surface) salt solution. The first 10 ns of instantaneous data are not used for calculation of cumulative averages. (TIF) [file pone.0027665.s002.tif]

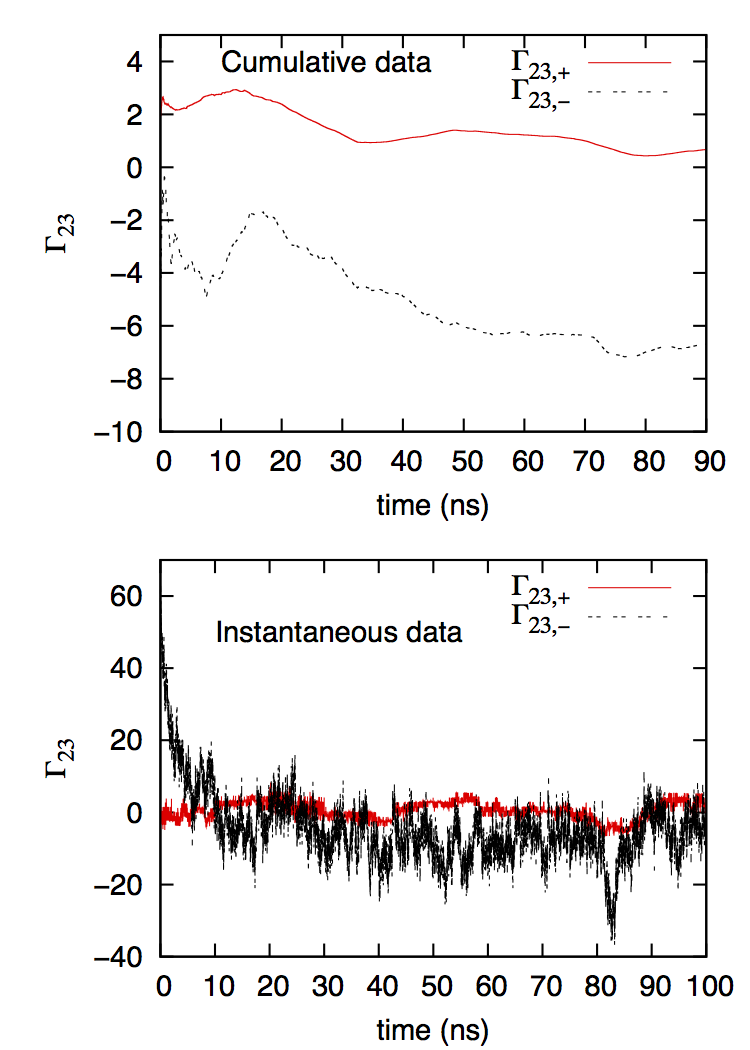

Supplement: Figure S2 — The influence of generation 0 PAMAM dendrimers, with surfaces modified to guanidinium, on aCgn monomer loss due to aggregation at 52.5°C. The figure depicts aCgn monomer concentration, M, versus time relative to the initial monomer concentration, M0, of 10 mg/mL, with all solutions prepared in a 20 mM sodium citrate pH 5 buffer and all profiles fitted to a 2nd order rate law. (A) Monomer loss profiles for solutions containing the guanidinium chloride salt form at varying concentrations. (B) Monomer loss profile for a solution containing the guanidinium dihydrogen phosphate salt form at a concentration of 0.2 M. The profiles for a solution containing no additive and a solution containing arginine hydrochloride at a concentration of 0.67 M are included for comparison. (TIFF) [file pone.0027665.s003.tif]

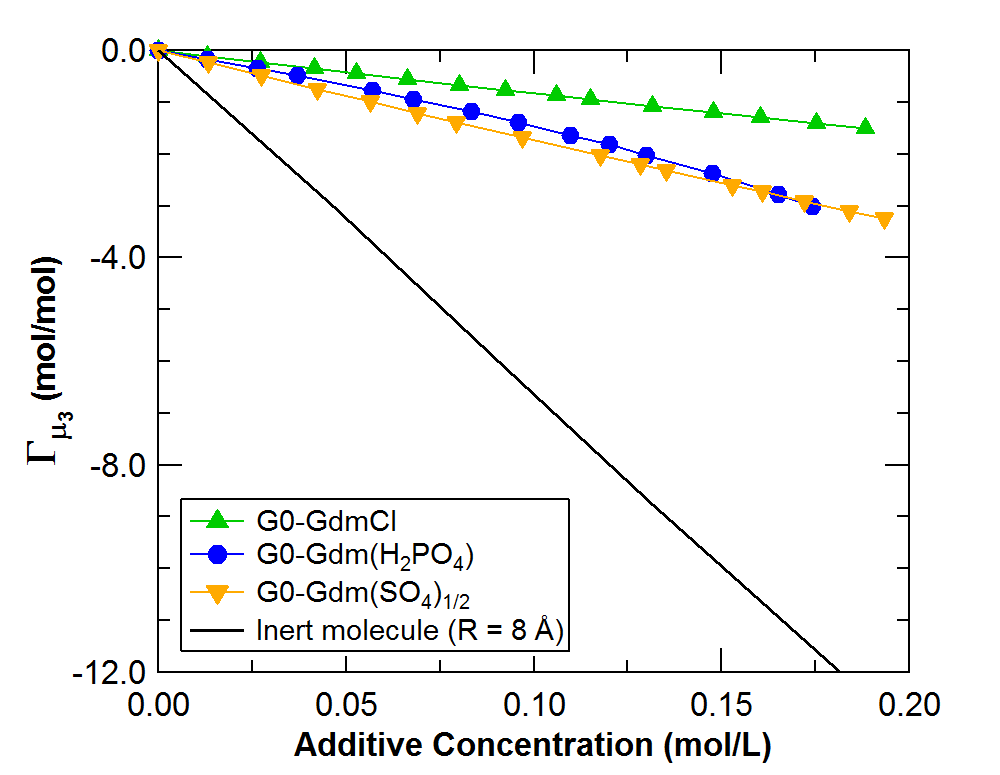

Supplement: Figure S3 — Preferential Interaction Coefficient, Γ μ 3 , values versus additive concentration. Values are for the interaction between generation 0 PAMAM dendrimers, with surfaces modified to guanidinium, and aCgn, as determined from VPO measurements. Error bars left off for clarity and curves drawn through the plots to aid the eye (see Table S2 for more detail). (TIF) [file pone.0027665.s004.tif]

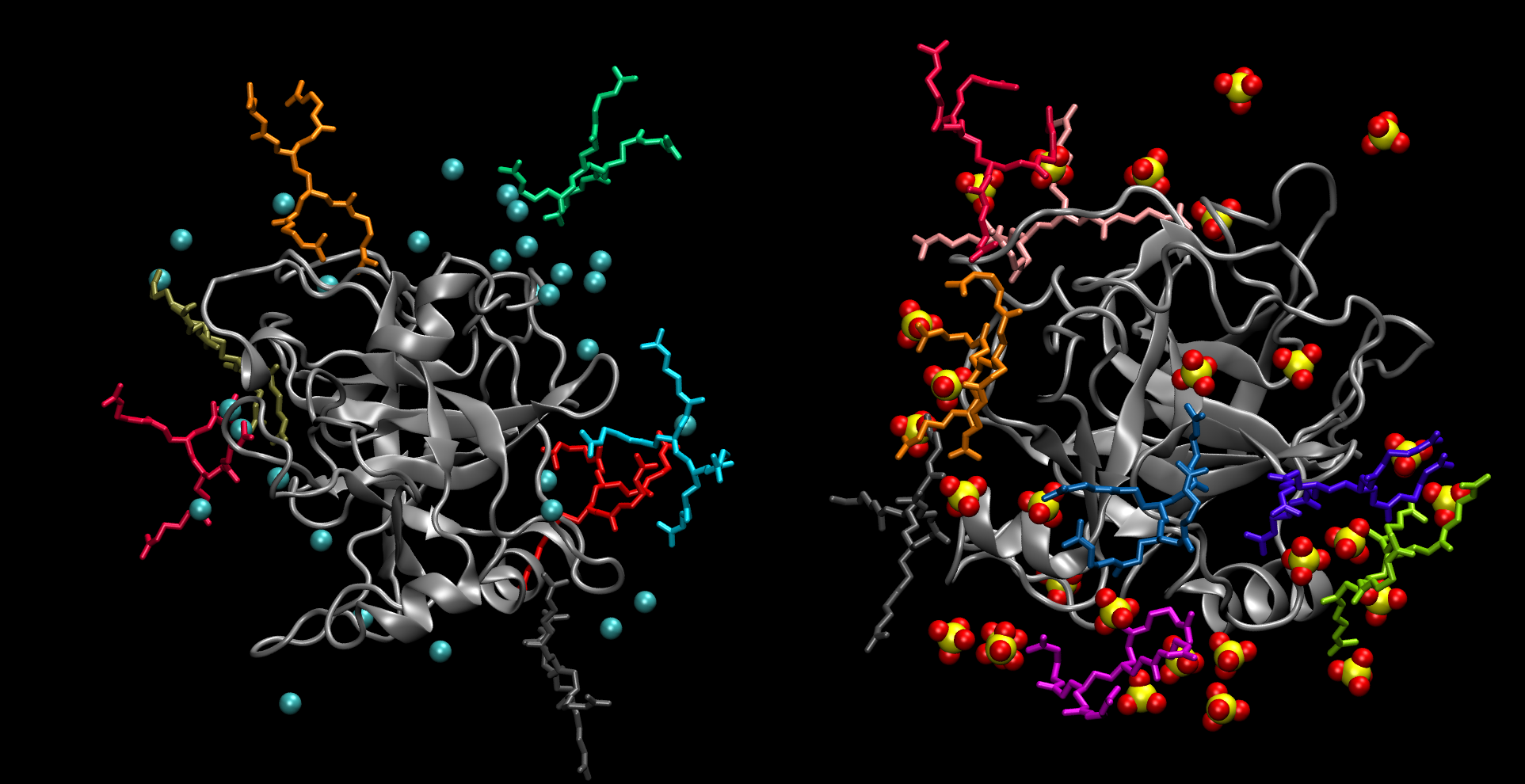

Supplement: Figure S4 — Snapshots of PAMAM dendrimer and counter-ions within 0.6 nm of the protein surface. Guanidinium chloride surface (left) and Guanidinium sulfate surface (right). (TIF) [file pone.0027665.s005.tif]
