# Supplementary material for: Effects of Solute-Solute Interactions on Protein Stability Studied Using Various Counterions and Dendrimers
Source: PLoS One. 2011 Nov 18;6(11):e27665. doi: 10.1371/journal.pone.0027665 (PMC3220676; doi:10.1371/journal.pone.0027665)
Supplement: Table S1 — Setup of simulation systems. (DOCX) [file pone.0027665.s006.docx]

**Table S1. Setup of simulation systems.**

| Simulation # | Surface | Protein | # of dendrimer | # of counter-ions | # of water molecules |
| --- | --- | --- | --- | --- | --- |
| S1 | GdmCl | - | 15 | 90 | 3889 |
| S2 | Gdm(SO_4_)_1/2_ | - | 15 | 45 | 3889 |
| S3 | Gdm(H_2_PO_4_) | - | 15 | 90 | 3889 |
| S4 | GdmCl | α-Cgn A | 36 | 222 | 10000 |
| S5 | Gdm(SO_4_)_1/2_ | α-Cgn A | 36 | 111 | 10000 |
| S6 | Gdm(H_2_PO_4_) | α-Cgn A | 36 | 222 | 10000 |

The molality of dendrimer salt solutions was set to 0.2 in all simulations.
