# Supplementary material for: Effects of Solute-Solute Interactions on Protein Stability Studied Using Various Counterions and Dendrimers
Source: PLoS One. 2011 Nov 18;6(11):e27665. doi: 10.1371/journal.pone.0027665 (PMC3220676; doi:10.1371/journal.pone.0027665)
Supplement: Table S2 — Summary of additive molecular weight, partial molar volume (V), preferential interactions with aCgn as determined by VPO, and aCgn denaturation midpoint temperature increment as determined by DSC for surface modified PAMAM dendrimers. (DOCX) [file pone.0027665.s007.docx]

Table S2: Summary of additive molecular weight, partial molar volume (V), preferential interactions with aCgn as determined by VPO, and aCgn denaturation midpoint temperature increment as determined by DSC for surface modified PAMAM dendrimers.

| Gen. | Surface | MW | V | Γ_23_ | dT_m_/d[3] | Max. [3] |
| --- | --- | --- | --- | --- | --- | --- |
|  |  | g/mol | L/mol | (mol/mol) | K*L/mol | mol/L |
| 0 | GdmCl | 903.6 | 0.5217+ 0.1499m | (-8.1 ± 3.6)[3] | -13.9 | 0.2 |
| 0 | Gdm(SO_4_)_1/2_ | 979.1 | 0.5665+ 0.1637m | (-17.0 ± 4.4)[3] | 15.2 | 0.2 |
| 0 | Gdm(H_2_PO_4_) | 1272.8 | 0.7254+ 0.1996m | (-15.8 ± 5.0)[3] | 37.4 | 0.2 |
| 1 | Gdm(H_2_PO_4_) | 3138.1 | 1.797+ 1.135m | - | - | - |
| 2 | Gdm(H_2_PO_4_) | 6868.7 | 3.819 + 11.39m | - | - | - |

The MW values represent the case for when all interior amines and surface groups are protonated and contain a counterion, m represents the molal concentration (mol/kg) of the additive, and [3] represents the molar concentration of the additive. Partial molar volume was determined from density measurements. aCgn solutions for the Γ_23_ (50 mg/mL) and T_m_ (1 mg/mL) data contained 20 mM sodium citrate pH 5 buffer. Solutions for the partial molar volume data contained no buffering component and the pH was not adjusted.
